# Photocatalytic H_2_ Evolution on TiO_2_ Assembled with Ti_3_C_2_ MXene and Metallic 1T-WS_2_ as Co-catalysts

**DOI:** 10.1007/s40820-019-0339-0

**Published:** 2019-12-16

**Authors:** Yujie Li, Lei Ding, Shujun Yin, Zhangqian Liang, Yanjun Xue, Xinzhen Wang, Hongzhi Cui, Jian Tian

**Affiliations:** School of Materials Science and Engineering, Shandong University of Science and Technology, Qingdao, 266590 People’s Republic of China

**Keywords:** Photocatalytic H_2_ production, Ti_3_C_2_ MXene, Octahedral phase WS_2_, TiO_2_ nanosheets, Co-catalysts

## Abstract

The biggest challenging issue in photocatalysis is efficient separation of the photoinduced carriers and the aggregation of photoexcited electrons on photocatalyst’s surface. In this paper, we report that double metallic co-catalysts Ti_3_C_2_ MXene and metallic octahedral (1T) phase tungsten disulfide (WS_2_) act pathways transferring photoexcited electrons in assisting the photocatalytic H_2_ evolution. TiO_2_ nanosheets were in situ grown on highly conductive Ti_3_C_2_ MXenes and 1T-WS_2_ nanoparticles were then uniformly distributed on TiO_2_@Ti_3_C_2_ composite. Thus, a distinctive 1T-WS_2_@TiO_2_@Ti_3_C_2_ composite with double metallic co-catalysts was achieved, and the content of 1T phase reaches 73%. The photocatalytic H_2_ evolution performance of 1T-WS_2_@TiO_2_@Ti_3_C_2_ composite with an optimized 15 wt% WS_2_ ratio is nearly 50 times higher than that of TiO_2_ nanosheets because of conductive Ti_3_C_2_ MXene and 1T-WS_2_ resulting in the increase of electron transfer efficiency. Besides, the 1T-WS_2_ on the surface of TiO_2_@Ti_3_C_2_ composite enhances the Brunauer–Emmett–Teller surface area and boosts the density of active site.

**Electronic supplementary material:**

The online version of this article (10.1007/s40820-019-0339-0) contains supplementary material, which is available to authorized users.

## Highlights

- The 1T-WS_2_@TiO_2_@Ti_3_C_2_ photocatalyst is highly active for water splitting to produce hydrogen at 3409.8 μmol g^−1^ h^−1^.
- The Ti_3_C_2_ MXene and octahedral (1T) phase WS_2_ act pathways transferring photogenerated electrons.

**Electronic supplementary material:**

The online version of this article (10.1007/s40820-019-0339-0) contains supplementary material, which is available to authorized users.

## Graphical abstract

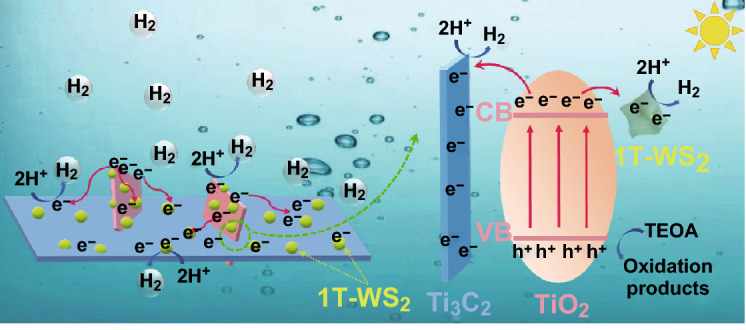

## Introduction

Due to energy consumption and consequent environmental pollution, the generation of hydrogen (H_2_) from water using solar light through semiconductors materials has aroused great attention [1–4]. Among these, TiO_2_ is widely studied owing to nontoxicity and low cost [5, 6]. However, the fast photoexcited carrier recombination restricts the TiO_2_’s application, and thus numerous efforts, such as doping, co-catalyst loading and heterostructure designing, are made to improve photoexcited carrier separation [7, 8]. Among these, co-catalysts can gather carriers to improve separation and act as active sites for H_2_ production [9]. Noble metals as excellent co-catalysts have widely applied to photocatalysis. However, extreme scarcity and high price restrict their application of photocatalytic water splitting [10, 11]. Therefore, seeking an inexpensive and highly active co-catalyst is of paramount significance for achieving photocatalytic H_2_ production in the future [12].

MXenes, as new 2D materials, have aroused remarkable attention because of its excellent electrical conductivity [13, 14]. For example, a 2D material with an accordion-like structure of layered Ti_3_C_2_ MXene can be prepared by etching Al layers from Ti_3_AlC_2_, in HF solution [15–17]. Due to its high electrical conductivity and unique layer morphology, Ti_3_C_2_ MXene is an appropriate substitute co-catalyst for noble metals for photocatalytic H_2_ evolution [18].

In recent years, transition metal disulfides (TMDs), such as molybdenum disulfide (MoS_2_) and tungsten disulfide (WS_2_), are regarded as promising substitutes for noble metals on catalysis [19, 20]. MoS_2_ and WS_2_ mainly include semiconductive trigonal (2H) phase and metallic octahedral (1T) phase [21, 22]. Both experimental and theoretical research have revealed that the metallic 1T phase possesses outstanding conductivity and more active sites, which will be suitable co-catalyst for photocatalytic H_2_ evolution, compared with 2H phase [23]. As one of the most popular TMDs materials, 1T phase MoS_2_ has been widely studied on photocatalysis [24–26]. However, the report about 1T phase WS_2_ (1T-WS_2_) on photocatalytic H_2_ production is still rare.

In this paper, an innovative 2D heterojunction by utilizing the metallic feature of Ti_3_C_2_ MXene and 1T-WS_2_ is reported. A two-step hydrothermal method is used for designing the novel 1T-WS_2_@TiO_2_@Ti_3_C_2_ photocatalyst where Ti_3_C_2_ MXene and 1TWS_2_ play important roles as electron acceptors. Firstly, TiO_2_ nanosheets are in situ grown on the surface of highly conductive Ti_3_C_2_ MXenes to construct TiO_2_@Ti_3_C_2_ composites by a facile hydrothermal method. Secondly, we intentionally employ the 1T-WS_2_ nanoparticles evenly distribute on TiO_2_@Ti_3_C_2_ composites’ surface using a hydrothermal process. This procedure results in the construction of an efficient photocatalytic system with intimate contact among metallic Ti_3_C_2_ MXene, 1T-WS_2_ nanoparticles, and TiO_2_ NSs. The newly designed 1T-WS_2_@TiO_2_@Ti_3_C_2_ composites exhibit extremely enhanced photocatalytic H_2_ evolution activity and stability owing to the novel structure.

## Experimental Procedures

### Materials

Ti_3_AlC_2_ powder was purchased from 11 Technology. Hydrochloric acid (HCl), sodium tetrafluoroborate (NaBF_4_), hydrofluoric acid (HF, 40 wt%), tungsten chloride (WCl_6_), thioacetamide (TAA), and dimethylformamide (DMF) were provided by Sinopharm.

### Synthesis of Ti_3_C_2_ MXenes

In a typical synthesis, 1 g Ti_3_AlC_2_ powders were dissolved in 120 mL HF solution (40 wt%) and were stirred for 72 h. Then, the mixed solution was washed with deionized (DI) water to neutral. Lastly, Ti_3_C_2_ MXenes were dried at 50 °C for overnight in a vacuum oven.

### Synthesis of TiO_2_@Ti_3_C_2_ Composites

Ti_3_C_2_ MXenes (400 mg) and NaBF_4_ (660 mg) were dissolved in 60 mL HCl (1.0 M) and were stirred for 30 min. The mixed solution was hydrothermally treated at 160 °C for 12 h. The obtained TiO_2_@Ti_3_C_2_ composites were washed with DI water and dried at 60 °C for overnight in a vacuum oven.

### Synthesis of 1T-WS_2_@TiO_2_@Ti_3_C_2_ Composites

WCl_6_ (24 mg) and TAA (9 mg) were added into 50 mL DMF. Then, 100 mg TiO_2_@Ti_3_C_2_ composites were dispersed in above solution and were stirred for 60 min. The mixed solution was hydrothermally treated at 200 °C for 24 h. The obtained 1T-WS_2_@TiO_2_@Ti_3_C_2_ composites (15 wt% WS_2_) were washed with DI water and dried at 60 °C for overnight in a vacuum oven. By adjusting the adding amount of WCl_6_ (16, 32, and 40 mg) and TAA (6, 12, and 15 mg), 1T-WS_2_@TiO_2_@Ti_3_C_2_ composites with other WS_2_ adding amounts (10, 20, and 25 wt%) were prepared, respectively.

### Characterizations

The phases of the samples were carried out using D/Max 2500PC X-ray diffraction (XRD). The surface characteristic and structure of the samples were tested by a FEI Nano 450 high-resolution scanning electron microscope (FESEM) and a JEOL 2100F high transmission electron microscope (HRTEM). The chemical states of the products were analyzed by a Thermo ESCALAB 250XI X-ray photoelectron spectrometry (XPS). The specific surface area and pore size distribution were tested by a nitrogen adsorption–desorption apparatus (Micromeritics ASAP2020) using the Brunauer–Emmett–Teller (BET) method. The UV–Vis diffuse reflectance spectra (DRS) of the products were measured using a Hitachi UH3101 UV–Vis spectrophotometer. The photoluminescence (PL) spectra were tested by a FLS920 fluorescence.

### Photoelectrochemical and Photocatalytic Activity Test

The photocatalytic activity test was measured using a Pyrex glass vessel, with a 300 W Xe arc lamp (CELHXF300) with an AM-1.5 filter as the light source. 10 mg of catalysts were added into acetone/TEOA solution (15 mL acetone + 5 mL TEOA + 80 mL DI water). The amount of generated H_2_ was tested by a gas chromatograph (Techcomp GC-7920). The electrochemical impedance spectroscopy (EIS) and transient photocurrent response (PEC) of the catalysts were measured by an electrochemical workstation (CHI660D) under a 300 W Xe arc lamp with an AM-1.5 filter in a three-electrode cell (0.5 M Na_2_SO_4_). Ag/AgCl electrode and Pt wire were used as reference and counter electrodes, respectively.

## Results and Discussion

**Scheme 1:**
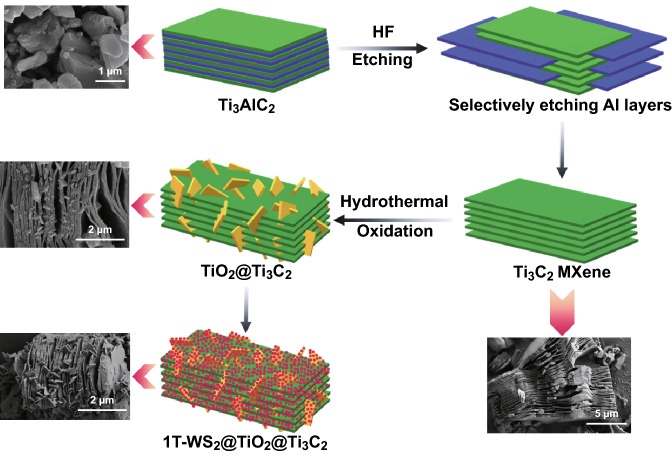
Schematic illustration of the preparation of 1T-WS_2_@TiO_2_@Ti_3_C_2_ composites

A typical synthesis route of 1T-WS_2_@TiO_2_@Ti_3_C_2_ composites is schematically depicted in Scheme 1. Ti_3_C_2_ MXenes are firstly prepared by etching Al layers of Ti_3_AlC_2_ MAX phase in HF solution [27]. Then, the layered Ti_3_C_2_ MXene provides Ti sources with the help of HCl and NaBF_4_ for growing TiO_2_ NSs across the layered Ti_3_C_2_ MXene. Finally, the obtained TiO_2_@Ti_3_C_2_ composites are added into WCl_6_/TAA solutions at 200 °C for 24 h to introduce 1T-WS_2_ co-catalysts. In this process, due to the intercalation of NH_4_^+^ of TAA, the space distance of WS_2_ increases and 1T-WS_2_ is generated [19]. The 1T-WS_2_ is evenly assembled on TiO_2_@Ti_3_C_2_ composites’ surface to construct the ternary 1T-WS_2_@TiO_2_@Ti_3_C_2_ composites.

**Fig. 1:**
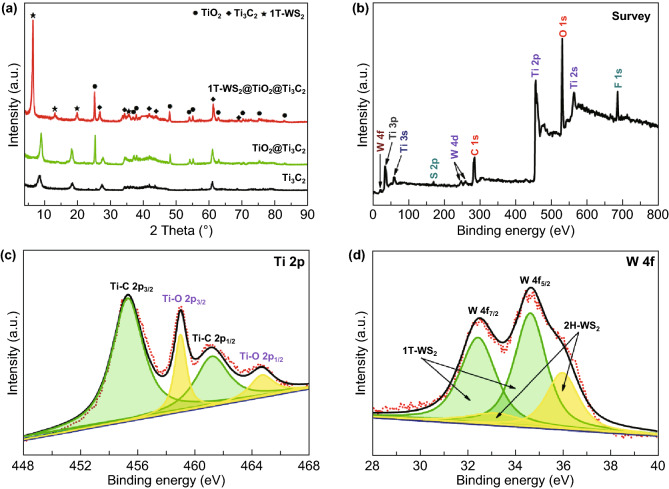
**a** XRD patterns of Ti_3_C_2_, TiO_2_@Ti_3_C_2_, and 1T-WS_2_@TiO_2_@Ti_3_C_2_ composites (15 wt% WS_2_); **b** fully scanned XPS spectrum, **c** Ti 2*p*, and **d** W 4*f* XPS spectra in 1T-WS_2_@TiO_2_@Ti_3_C_2_ composites (15 wt% WS_2_)

After HF etching, the most intense XRD (104) peak of Ti_3_AlC_2_ was disappeared, and the (002) peak of Ti_3_AlC_2_ at 9.52^o^ was moved to lower 2-theta value (8.78^o^), which indicates the successful formation of Ti_3_C_2_ (Fig. S1a) [17]. The development of TiO_2_ nanosheets across Ti_3_C_2_ MXenes by the hydrothermal oxidation of Ti_3_C_2_ is evidenced by the emergence of diffraction peaks of anatase TiO_2_ (JCPDS No. 21-1272) as shown in Fig. 1a. The XRD peaks appearing at 6.66°, 13.40°, and 20.10° are indexed to (002), (004), and (006) planes of 1T-WS_2_ [23]. The co-existence of Ti_3_C_2_, TiO_2_, and 1T-WS_2_ indicates the successful preparation of 1T-WS_2_@TiO_2_@Ti_3_C_2_ composites. For 1T-WS_2_@TiO_2_@Ti_3_C_2_ composites with other WS_2_ ratios (Fig. S1b), all the XRD peaks are well corresponding to Ti_3_C_2_, TiO_2_, or 1T-WS_2_.

The full-scale XPS spectrum of 1T-WS_2_@TiO_2_@Ti_3_C_2_ composites (Fig. 1b) displays that Ti, C, O, S, and W are dominant elements, while F element is ascribed to F^−^ ions physically adsorbed on composites from the HF solution. The Ti 2*p* spectrum is divided into four peaks (Fig. 1c). The two peaks at 464.7 (Ti–O 2*p*_1/2_) and 459.0 eV (Ti–O 2*p*_3/2_) are ascribed to lattice Ti–O bonds of TiO_2_ [28]. The other two peaks at 461.2 (Ti-C 2*p*_1/2_) and 455.3 eV (Ti-C 2*p*_3/2_) are indexed to lattice Ti–C bonds of Ti_3_C_2_ [29]. The high-resolution Ti 2*p* XPS spectrum indicates the content of Ti_3_C_2_ is about 79% in TiO_2_@Ti_3_C_2_ composites. The W 4*f* XPS spectrum of 1T-WS_2_@TiO_2_@Ti_3_C_2_ composites (Fig. 1d) can confirm the presence and relative content of 1T-WS_2_. In the W 4*f* region, the two peaks of 2H phase corresponding to W 4*f*_7/2_ and W 4*f*_5/2_ at 33.0 and 36.0 eV, respectively. Nevertheless, two extra peaks shift to lower binding energies at 32.4 and 34.6 eV, suggesting the existence of 1T-WS_2_ [19, 30]. The 1T phase content is calculated about 73%, which shows that the 1T-WS_2_@TiO_2_@Ti_3_C_2_ composites are composed of lots of metallic 1T phase.

**Fig. 2:**
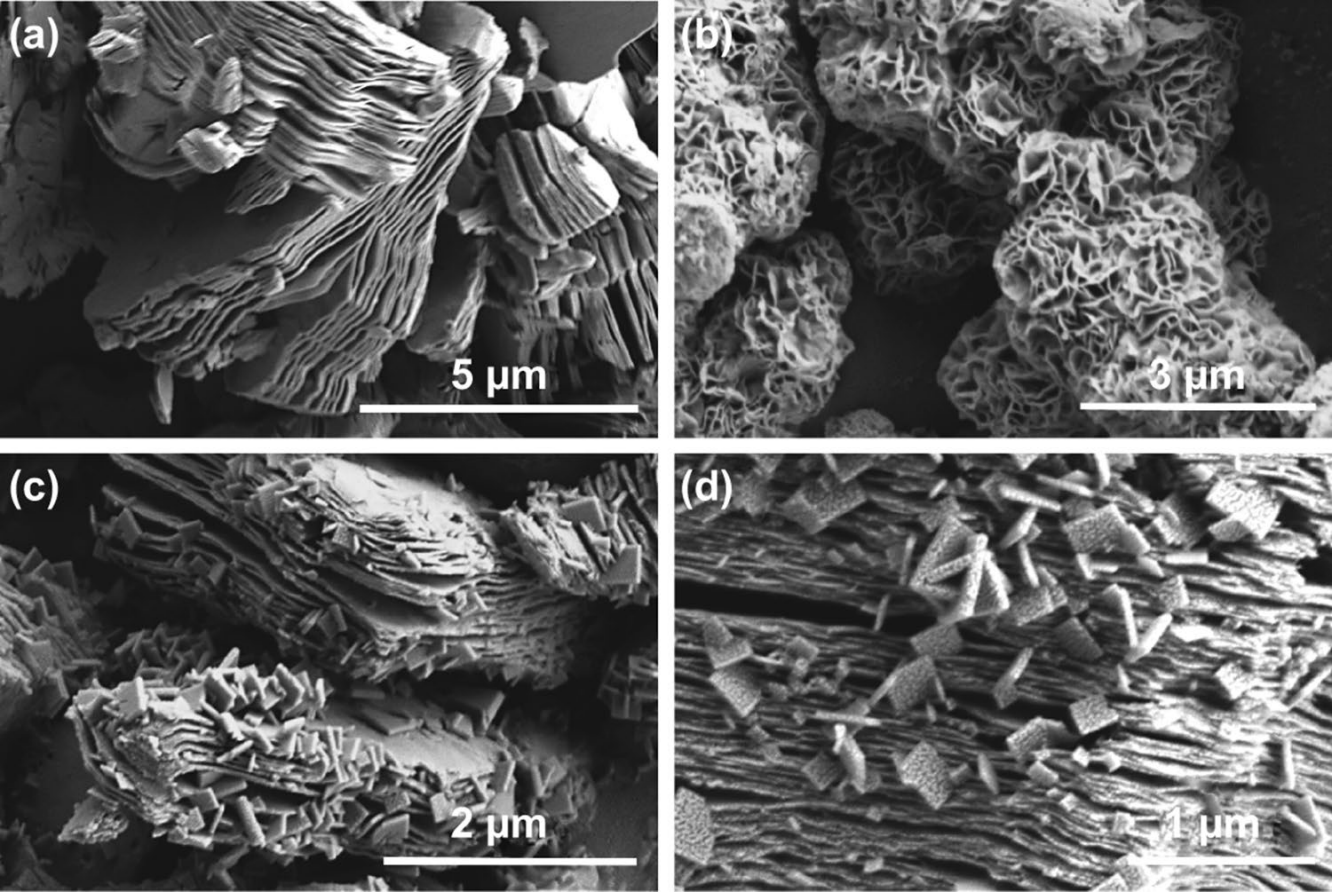
SEM images of **a** Ti_3_C_2_ MXene, **b** 1T-WS_2_, and **c, d** 1T-WS_2_@TiO_2_@Ti_3_C_2_ composites (15 wt% WS_2_)

Ti_3_C_2_ MXenes are obtained by etching of the aluminum layer of the bulk Ti_3_AlC_2_ (Fig. S2a) by using HF. As shown in Fig. 2a, Ti_3_C_2_ MXenes present typical accordion-like multilayer structure. After hydrothermal oxidation of Ti_3_C_2_ MXenes, the layered Ti_3_C_2_ MXenes provide Ti sources for growing TiO_2_ NSs inserting across the layered Ti_3_C_2_ MXene to form TiO_2_@Ti_3_C_2_ composites, and further combine with WS_2_ through hydrothermal reaction to get 1T-WS_2_@TiO_2_@Ti_3_C_2_ composites. Figure 2b shows that 1T-WS_2_ presents nanoflake structures and agglomerate into nanoflowers. After combining with TiO_2_@Ti_3_C_2_ composites, the 1T-WS_2_ nanoparticles are evenly distributed on TiO_2_@Ti_3_C_2_ composites’ surface (Fig. 2c, d). Furthermore, 1T-WS_2_@TiO_2_@Ti_3_C_2_ composites with other WS_2_ ratios (10, 20, and 25 wt%) are prepared, and corresponding SEM images are displayed in Fig. S2.

**Fig. 3:**
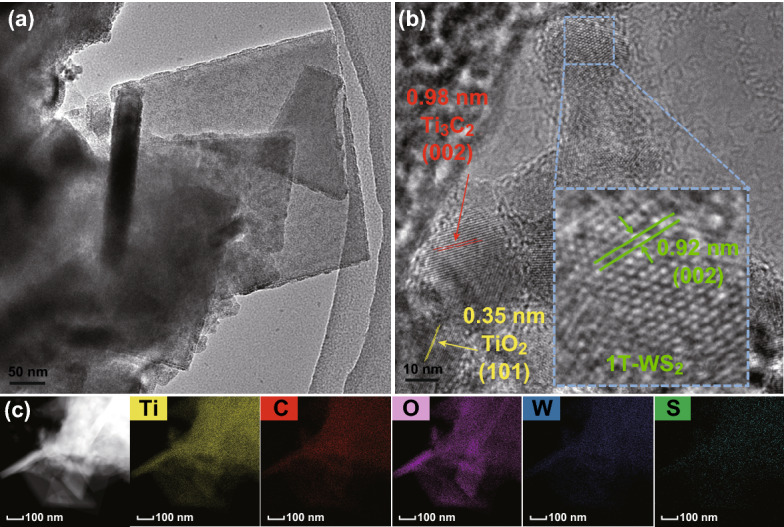
**a**, **b** HRTEM and **c** EDX elemental mapping images of 1T-WS_2_@TiO_2_@Ti_3_C_2_ composites (15 wt% WS_2_)

The phase composition and microscopic structure of 1T-WS_2_@TiO_2_@Ti_3_C_2_ composites are characterized by TEM (Fig. 3a, b). The lattice with *d* spaces of 0.35 nm is attributed to (101) plane of anatase TiO_2_ (Fig. 3b), which are same as the description of the literatures [31, 32]. The lattice spacings of 0.98 and 0.92 nm are indexed to (002) plane of Ti_3_C_2_ MXene and (002) plane of 1T-WS_2_ [19, 33–35] (Fig. 3b). The EDX element mappings of composites (Fig. 3c) indicate that the Ti, C, O, W, and S elements are accordantly distributed. The as-fabricated photocatalyst with superior metallic quality of Ti_3_C_2_ MXene and 1T-WS_2_ present more effective carrier transfer and separation compared with TiO_2_ NSs, and therefore, the photocatalytic performance is enhanced. The 1T-WS_2_ is further confirmed by Raman spectroscopy (Fig. S3). Remarkably, in contrast to 2H phase WS_2_, there are no scattering peaks between 350 and 450 cm^−1^ attributed to $E_{2g}^{1}$ (in-plane) and *A*_*1g*_ (out-of-plane) in 1T-WS_2_ (Fig. S3). There are also two strong peaks at low frequency range for 1T-WS_2_. One strong Raman band at 128 cm^−1^ (*J*_1_) is attributed to W–W stretching vibrations in 1T-WS_2_@TiO_2_@Ti_3_C_2_ composite [21]. Besides, another additional peak at 171 cm^−1^ (*J*_2_) is observed, which is associated with the phonon modes in the WS_2_, suggesting the existence of a considerable amount of 1T phase ingredient embedded [22]. This result further implies that the as-prepared WS_2_ in 1T-WS_2_@TiO_2_@Ti_3_C_2_ composites is mostly 1T phase [19, 23].

**Fig. 4:**
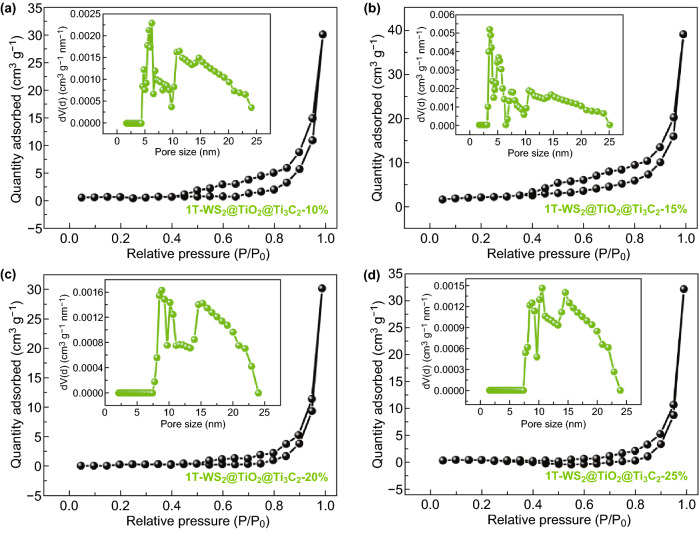
N_2_ adsorption–desorption isotherms and corresponding pore size distribution curves (inset) of 1T-WS_2_@TiO_2_@Ti_3_C_2_ composites with **a** 10 wt%, **b** 15 wt%, **c** 20 wt%, and **d** 25 wt% WS_2_ ratios

To further examine the textural properties of 1T-WS_2_@TiO_2_@Ti_3_C_2_ composites, the isotherms and the pore size distributions are studied by N_2_ adsorption–desorption measurement (Figs. 4 and S4). All of samples present type IV isotherms with H3 hysteresis loops, suggesting the presence of mesopores [36]. And the pore size distribution curves of 1T-WS_2_@TiO_2_@Ti_3_C_2_ composites with different WS_2_ loading amounts (Fig. 4a–d inset) display that the size of major mesopores ranges from 2 to 25 nm. Compared with other samples, when the loading amount of WS_2_ is 15%, the pore size distribution is relatively concentrated at 2 ~ 5 nm. The presence of such a small pore size is conducive to migration of reactant and product molecules to facilitate photocatalytic reactions. Moreover, larger nitrogen adsorption capacity indicates that more reactive sites may be provided during the reaction process, which is favorable in the enhancement of catalytic activity. The BET surface area of as-prepared 1T-WS_2_@TiO_2_@Ti_3_C_2_-15%, as shown in Table S1, reveals a higher surface area (23.334 m^2^ g^−1^) than those of Ti_3_C_2_ MXene, pure 1T-WS_2_, and 1T-WS_2_@TiO_2_@Ti_3_C_2_ composites with other WS_2_ loading amounts. In addition, as shown in Figs. 4 and S4, the BET surface area of the samples increase nonlinearly with increasing WS_2_ loading. As the WS_2_ loading amount increases from 10 to 15 wt%, the BET surface area of composites increases. However, further increasing the loading amount of WS_2_ (from 15 to 25 wt% WS_2_) leads to a gradual decrease of BET surface area, which may be caused by the aggregation of WS_2_ on the surface of the composites. The higher surface areas are beneficial for photocatalysis since it could provide more adsorption and active sites, thus the photocatalytic activity is improved [37].

**Fig. 5:**
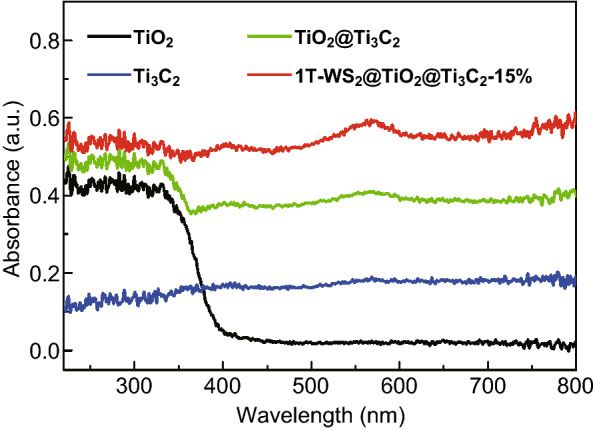
UV–Vis DRS spectra of Ti_3_C_2_ MXene, TiO_2_ NSs, TiO_2_@Ti_3_C_2_, and 1T-WS_2_@TiO_2_@Ti_3_C_2_ composites (15 wt% WS_2_)

To investigate the optical absorptivity, the UV–Vis DRS spectra of samples are measured. As shown in Fig. 5, TiO_2_ NSs (curve black) have a noticeable UV light absorption, due to the nature of anatase TiO_2_ [38]. Ti_3_C_2_ MXene (curve blue) shows UV and visible absorption as a result of the black color nature [39]. Compared with TiO_2_ NSs, 1T-WS_2_@TiO_2_@Ti_3_C_2_ composites (15 wt% WS_2_) display a significant absorption edge red shift and enhanced visible absorption, which is attributed to the optical absorption of Ti_3_C_2_ MXene and 1T-WS_2_. The increase of light absorption range of photocatalysts will be more helpful to promote the progress of photocatalytic reaction. Besides, the 1T-WS_2_@TiO_2_@Ti_3_C_2_ composites show stronger light absorption with the increase of WS_2_ contents from 10 to 25 wt% (Fig. S5).

**Fig. 6:**
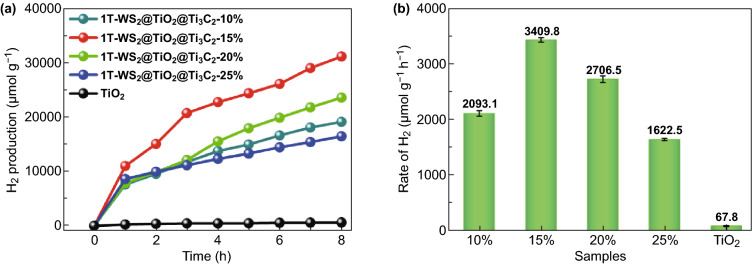
**a** Photocatalytic H_2_ production and **b** rate of the samples under simulated sunlight illumination

The photocatalytic performance of 1T-WS_2_@TiO_2_@Ti_3_C_2_ composites was evaluated using H_2_ evolution under simulated sunlight irradiation in an aqueous acetone solution at room temperature (Fig. 6). Control experiments (Fig. S6) show that no noticeable H_2_ evolution is discovered without either photocatalyst or illumination. TiO_2_ NSs (Fig. 6) present limited photocatalytic H_2_ activity (67.8 μmol g^−1^ h^−1^), arising from fast carrier recombination [40]. In view of the excellent electronic conductivity of Ti_3_C_2_ and 1T-WS_2_, it is combined as a co-catalyst with TiO_2_ NSs in order to achieve better photogenerated carrier separation and improve photocatalytic performance [41, 42]. As expected, after assembling of Ti_3_C_2_ and 1T-WS_2_, the bets photocatalytic activity is detected (3409.8 μmol g^−1^ h^−1^ for 1T-WS_2_@TiO_2_@Ti_3_C_2_ composites (15 wt% WS_2_)), which is nearly 50 times higher than that of TiO_2_ NSs. Moreover, the 1T-WS_2_@TiO_2_@Ti_3_C_2_ composites (10 wt% WS_2_) present a lower photocatalytic activity than 1T-WS_2_@TiO_2_@Ti_3_C_2_ composites (15 wt% WS_2_), owing to the relatively weaker solar light input. Furthermore, with the increase of WS_2_ contents from 15 to 25 wt%, a reduction in the photocatalytic performance of 1T-WS_2_@TiO_2_@Ti_3_C_2_ composites is discovered. Because excess black 1T-WS_2_ nanoparticles induced “shielding effect” block light to the surface of TiO_2_ [43].

Besides, we evaluate the apparent quantum efficiency (AQE) of photocatalysts under the same light source. Table S2 displays the comparison of AQE values of TiO_2_ NSs and 1T-WS_2_@TiO_2_@Ti_3_C_2_ composites with different WS_2_ ratios (10, 15, 20, and 25 wt%): 0.049% (TiO_2_ NSs) < 1.173% (1T-WS_2_@TiO_2_@Ti_3_C_2_-25 wt%) < 1.513% (1T-WS_2_@TiO_2_@Ti_3_C_2_-10 wt%) < 1.956% (1T-WS_2_@TiO_2_@Ti_3_C_2_-20 wt%) < 2.464% (1T-WS_2_@TiO_2_@Ti_3_C_2_-15 wt%), which is in accordance with photocatalytic H_2_ evolution performance. Moreover, we conduct stability of 1T-WS_2_@TiO_2_@Ti_3_C_2_ composites (15 wt% WS_2_) for 24 h (Fig. S7). No noticeable H_2_ production decrease is detected after 3 cycles (24 h). SEM images (Fig. S8) and XRD pattern (Fig. S9) of 1T-WS_2_@TiO_2_@Ti_3_C_2_ composites after 3 cycles display no evident difference compared with fresh samples. The results further demonstrate that 1T-WS_2_@TiO_2_@Ti_3_C_2_ composites can act as a favorable photocatalyst for H_2_ production. We also delaminated the multilayered Ti_3_C_2_ MXenes to get monolayered Ti_3_C_2_ nanosheets (Fig. S10). The XRD pattern of TiO_2_@Ti_3_C_2_ (monolayer) does not detect the diffraction peak of Ti_3_C_2_ (Fig. S10a). Since Ti_3_C_2_ monolayer is in full contact with the reaction solution, all Ti_3_C_2_ may be converted into TiO_2_ under the same experimental conditions. As shown in Fig. S10b, 1T-WS_2_@Ti_3_C_2_@Ti_3_C_2_ (monolayer) presents worse photocatalytic H_2_ production activity than that of 1T-WS_2_@TiO_2_@Ti_3_C_2_ (multilayer), which demonstrates that the lack of Ti_3_C_2_ by oxidation in 1T-WS_2_@Ti_3_C_2_@Ti_3_C_2_ (monolayer) greatly affects the photocatalytic H_2_ production. Furthermore, changing the ratio between Ti_3_C_2_ and TiO_2_ also affecting the photocatalytic performance of 1T-WS_2_@TiO_2_@Ti_3_C_2_ composites (Fig. S11). Compared with in situ loading of TiO_2_ nanosheets (Fig. S12), foreign titanium sources do not improve the photocatalytic performance of 1T-WS_2_@TiO_2_@Ti_3_C_2_ composites, which may be caused by the non-close contact between TiO_2_ and Ti_3_C_2_ caused by the foreign titanium sources.

**Fig. 7:**
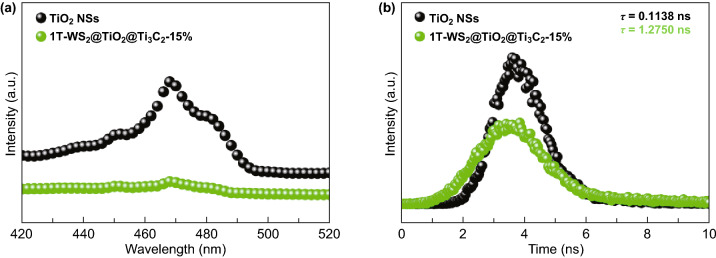
**a** Steady and **b** time-resolved PL spectra of TiO_2_ NSs and 1T-WS_2_@TiO_2_@Ti_3_C_2_ composites (15 wt% WS_2_), *λ*_ex_ = 325 nm

The introduction of Ti_3_C_2_ MXene and 1T-WS_2_ in 1T-WS_2_@TiO_2_@Ti_3_C_2_ composites would be believed to influence photoinduced carrier separation, which could be characterized by steady and time-resolved PL spectroscopy (Fig. 7). As illustrated in Fig. 7a, TiO_2_ NSs possess a high PL peak, resulting in the quick photoinduced carrier recombination. When Ti_3_C_2_ MXene and 1T-WS_2_ are incorporated, the PL peak is significantly reduced (Fig. 7a). Evidently, the photoinduced carrier recombination of TiO_2_ is hindered by migrating electrons to Ti_3_C_2_ and 1T-WS_2_ as electron acceptors [44]. An increased lifetime of charge carriers is also detected by loading Ti_3_C_2_ MXene and 1T-WS_2_ (Fig. 7b). The intensity-average lifetimes (*τ*) of TiO_2_ NSs are 0.1138 ns, much shorter than that of 1T-WS_2_@TiO_2_@Ti_3_C_2_ composites (1.2750 ns). The increased carrier lifetime of 1T-WS_2_@TiO_2_@Ti_3_C_2_ composites is beneficial for enhanced carrier separation efficiency.

**Fig. 8:**
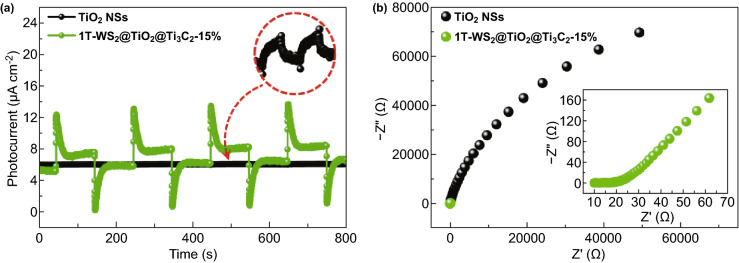
**a** Transient photocurrent responses and **b** EIS of TiO_2_ NSs and 1T-WS_2_@TiO_2_@Ti_3_C_2_ composites (15 wt% WS_2_)

The photocurrent responses of photocatalysts were prompt by some on–off cycles under light illumination (Fig. 8a). All of samples present reversible photocurrent responses on each irradiation. The photocurrent intensity of 1T-WS_2_@TiO_2_@Ti_3_C_2_ composites is much higher than that of pure TiO_2_ NSs, which is due to Ti_3_C_2_ and 1T-WS_2_ as co-catalysts more effectively receiving photoexcited electrons of TiO_2_. The 1T-WS_2_@TiO_2_@Ti_3_C_2_ composites (Fig. 8b) exhibit a smaller arc radius compared with TiO_2_ NSs under light irradiation, suggesting that the 1T-WS_2_@TiO_2_@Ti_3_C_2_ composite presents smaller charge transfer resistance, finally causing higher photoexcited carrier transfer and separation efficiency [45].

**Scheme 2:**
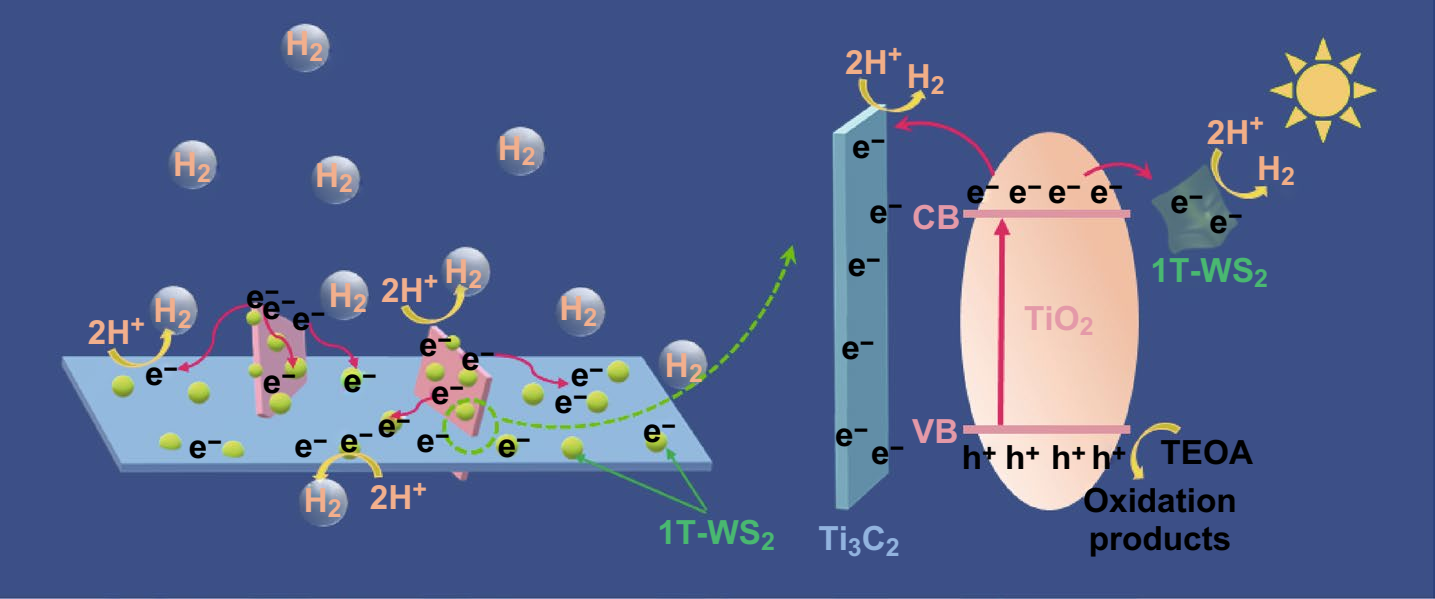
Schematic photocatalytic mechanism of 1T-WS_2_@TiO_2_@Ti_3_C_2_ composites

As shown in Scheme 2, under light irradiation, TiO_2_ NSs can be excited to produce electrons and holes. The majority of photoexcited electrons in conduction band (CB) of TiO_2_ could instantly migrate to metallic Ti_3_C_2_ MXene and 1T-WS_2_ through the interface. As the photoelectron receivers, Ti_3_C_2_ MXene and 1T-WS_2_ serves as active sites for H_2_ production [46, 47]. Meanwhile, the holes in the valence band (VB) of TiO_2_ are consumed by the sacrificial reagents. Consequently, the photoexcited carriers are efficiently transferred and separated with the assistance of double co-catalysts Ti_3_C_2_ and 1T-WS_2_.

## Conclusions

In conclusion, an effective 1T-WS_2_@TiO_2_@Ti_3_C_2_ composite photocatalyst is successfully prepared. The development of TiO_2_ NSs on Ti_3_C_2_ MXenes and 1T-WS_2_ nanoparticles uniformly distributing on TiO_2_@Ti_3_C_2_ composite is the design concept. The obtained 1T-WS_2_@TiO_2_@Ti_3_C_2_ composite with 15 wt% WS_2_ loading displays excellent photocatalytic H_2_ production performance (3409.8 μmol g^−1^ h^−1^), nearly 50 times higher than that of pure TiO_2_ NSs. The excellent H_2_ evolution performance of 1T-WS_2_@TiO_2_@Ti_3_C_2_ composites is ascribed to the following reasons: (1) The introduction of 1T-WS_2_ nanoparticles induces enhanced BET surface area and more active sites; (2) Both Ti_3_C_2_ MXene and 1T-WS_2_ possess extraordinary conductivity, which greatly enhance the electron transfer ability and thus achieve highly efficient spatial charge separation.

## Electronic supplementary material

Below is the link to the electronic supplementary material.

Supplementary material 1 (PDF 1051 kb)
