# Supplementary material for: Photocatalytic H2 Evolution on TiO2 Assembled with Ti3C2 MXene and Metallic 1T-WS2 as Co-catalysts
Source: Nanomicro Lett. 2019 Dec 16;12:6. doi: 10.1007/s40820-019-0339-0 (PMC7770731; doi:10.1007/s40820-019-0339-0)
Supplement: Supplementary file 1 — Supplementary material 1 (PDF 1051 kb) [file 40820_2019_339_MOESM1_ESM.pdf]

Supporting Information for

Photocatalytic H_2 Evolution on TiO_2 Assembled with Ti_3C_2 MXene and Metallic 1T-WS_2 as Cocatalysts

Yujie Li[1,#], Lei Ding[1,#], Shujun Yin[1], Zhangqian Liang[1], Yanjun Xue[1], Xinzhen Wang[1,*], Hongzhi Cui[1,*], Jian Tian[1,*]

[1]School of Materials Science and Engineering, Shandong University of Science and Technology, Qingdao 266590, People's Republic of China

*Corresponding authors. Email: xzwang@sdust.edu.cn (Xinzhen Wang); cuihongzhi1965@163.com (Hongzhi Cui); jiantian@sdust.edu.cn (Jian Tian)

[#]These authors contributed equally to this work.

Supplementary Figures

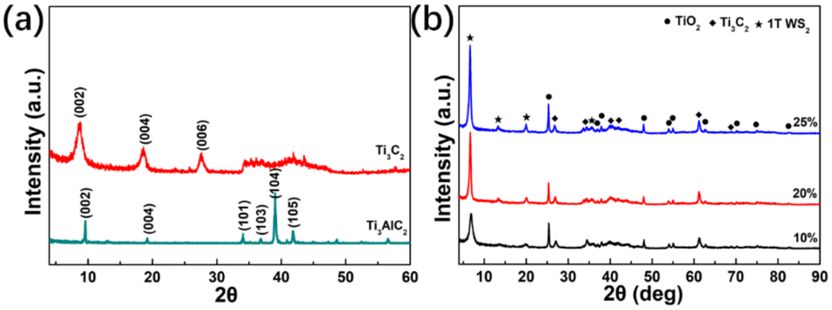

Fig. S1 XRD patterns of (**a**) Ti_3AlC_2, Ti_3C_2 MXene and (**b**) 1T-WS_2@TiO_2@Ti_3C_2 composites with different WS_2 loading amounts (10, 20, and 25 wt%)

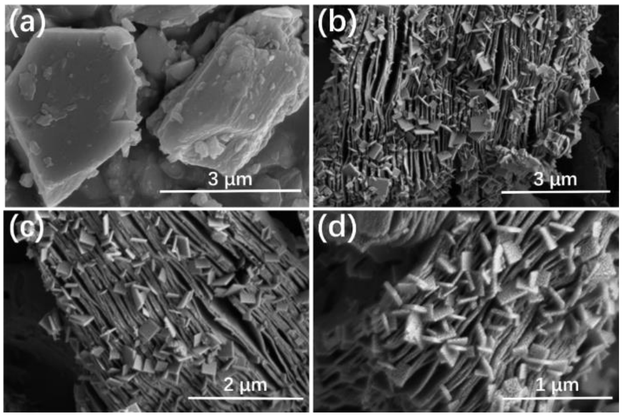

Fig. S2 SEM images of (**a**) Ti_3AlC_2 MAX and 1T-WS_2@TiO_2@Ti_3C_2 composites with different WS_2 loading amount (**b**) 10 wt%, (**c**) 20 wt%, and (**d**) 25 wt%

Table S1 BET surface area of Ti_3C_2 MXene, 1T-WS_2@TiO_2@Ti_3C_2 composites with different WS_2 loading amounts (10, 15, 20, and 25 wt% MoS_2) and the 1T-WS_2

**Samples**	**BET Surface area (m^2 g^{-1})**
Ti_3C_2 MXene	7.081
1T-WS_2@TiO_2@Ti_3C_2-10 wt%	21.105
1T-WS_2@TiO_2@Ti_3C_2-15 wt%	23.334
1T-WS_2@TiO_2@Ti_3C_2-20 wt%	22.001
1T-WS_2@TiO_2@Ti_3C_2-25 wt%	19.677
1T phase WS_2	6.302

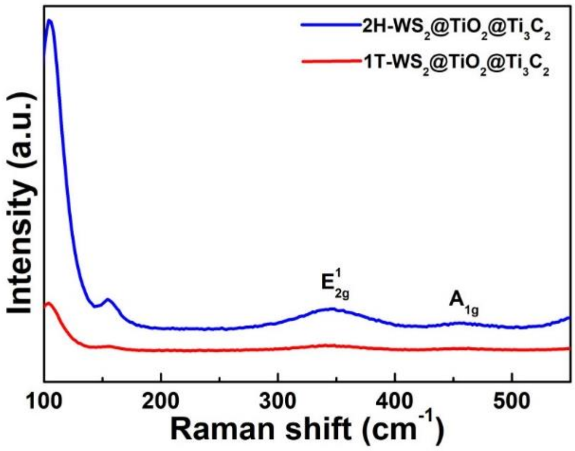

Fig. S3 Raman spectra of 2H-WS_2@TiO_2@Ti_3C_2 and 1T-WS_2@TiO_2@Ti_3C_2 composite

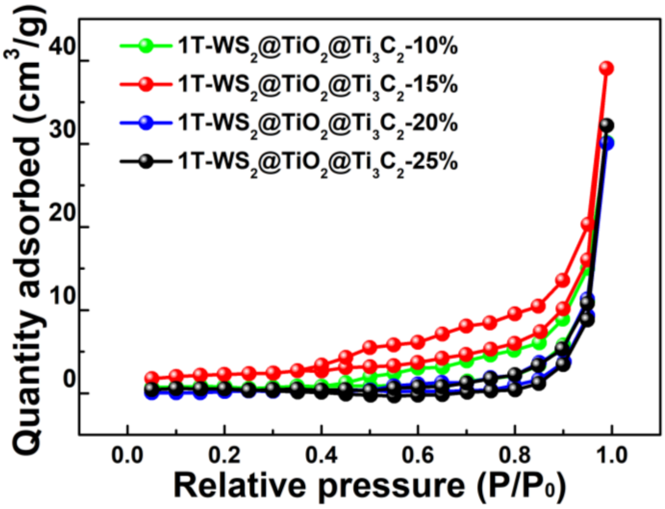

Fig. S4 N_2 adsorption–desorption isotherms curves of 1T-WS_2@TiO_2@Ti_3C_2 composites with 10, 15, 20, and 25 wt% WS_2 ratios

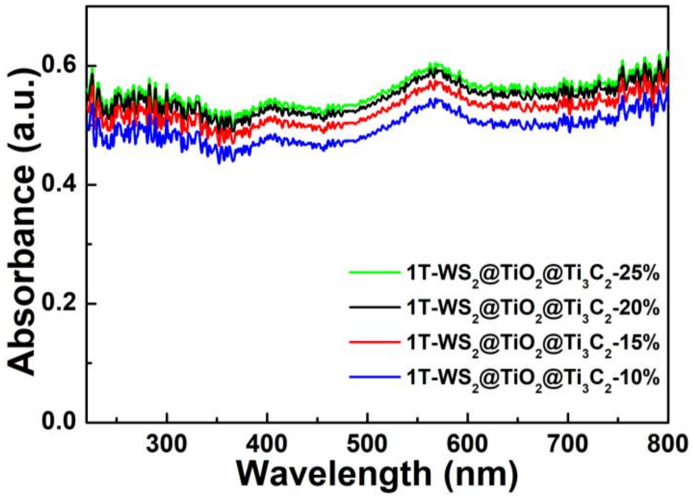

Fig. S5 UV–Vis diffuse reflectance spectra of 1T-WS_2@TiO_2@Ti_3C_2 composites with different WS_2 loading amounts (10, 15, 20, and 25 wt%)

Table S2 Comparison of AQE values over the photocatalysts

Sample	AQE values (%)
TiO_2 NSs	0.049%
1T-WS_2@TiO_2@Ti_3C_2-10 wt%	1.513%
1T-WS_2@TiO_2@Ti_3C_2-15 wt%	2.464%
1T-WS_2@TiO_2@Ti_3C_2-20 wt%	1.956%
1T-WS_2@TiO_2@Ti_3C_2-25 wt%	1.173%

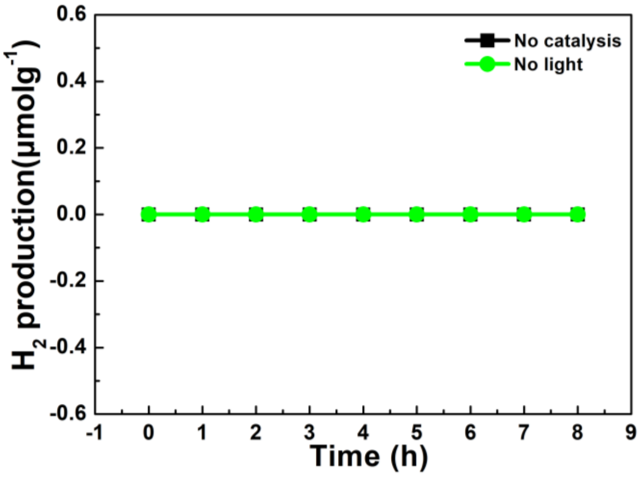

Fig. S6 Photocatalytic H_2 production of control experiments in the absence of irradiation and photocatalyst

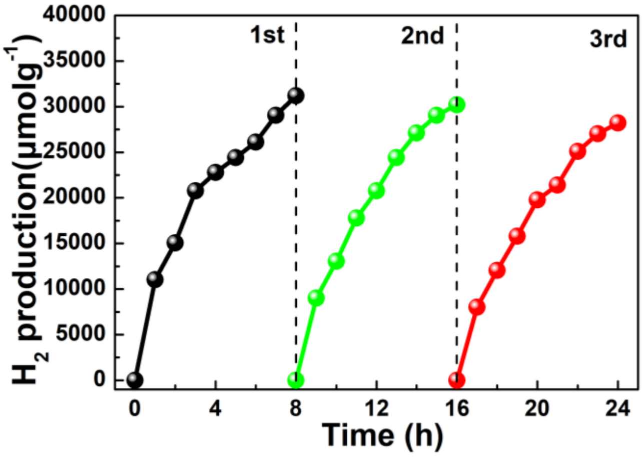

Fig. S7 Stability and recyclability of the 1T-WS_2@TiO_2@Ti_3C_2 composites (15 wt% WS_2)

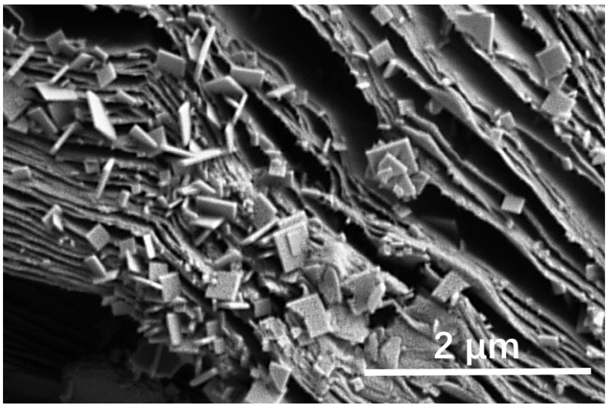

Fig. S8 SEM image of 1T-WS_2@TiO_2@Ti_3C_2 composites (15 wt% WS_2) after 3 cycles

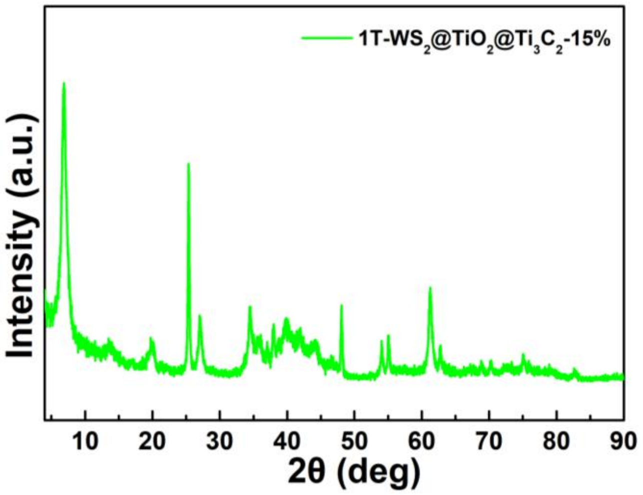

Fig. S9 XRD pattern of 1T-WS_2@TiO_2@Ti_3C_2 composites (15 wt% WS_2) after 3 cycles

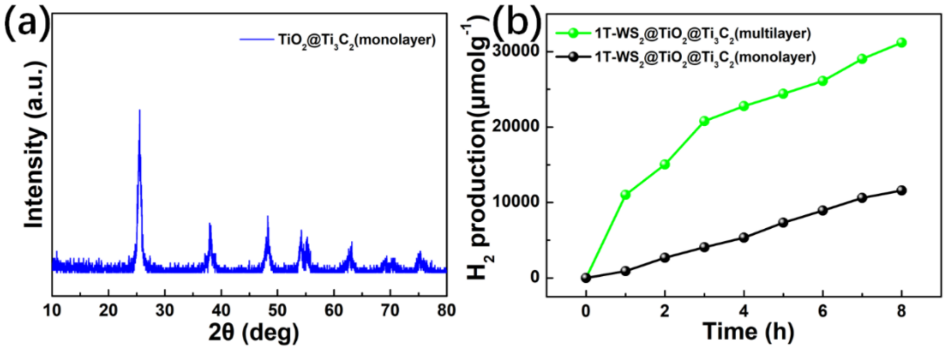

Fig. S10 (**a**) XRD patterns of TiO_2@Ti_3C_2 (monolayer) and (**b**) photocatalytic H_2 production of the samples under simulated sunlight illumination

We have delaminated the multilayered Ti_3C_2 MXenes to get monolayered Ti_3C_2 nanosheets (**Fig.** S10), and the resulting dispersion concentration is 2 mg mL^{-1}. In the process of preparing TiO_2@Ti_3C_2 (monolayer), the reaction conditions are guaranteed to be consistent with the preparation conditions of TiO_2@Ti_3C_2 (multilayer) in the article, that is, the amount of other materials and the reaction conditions remain unchanged. As shown in **Fig.** S10a, the XRD patterns of TiO_2@Ti_3C_2 (monolayer) did not detect the diffraction peak of Ti_3C_2. Since Ti_3C_2 monolayer is in full contact with the reaction solution, all Ti_3C_2 may be converted into TiO_2 under the same experimental conditions. As shown in **Fig.** S10b, 1T-WS_2@Ti_3C_2@Ti_3C_2 (monolayer) presents worse photocatalytic H_2 production activity than that of 1T-WS_2@TiO_2@Ti_3C_2 (multilayer), which demonstrate that the lack of Ti_3C_2 by oxidation in 1T-WS_2@Ti_3C_2@Ti_3C_2 (monolayer) greatly affects the photocatalytic H_2 production.

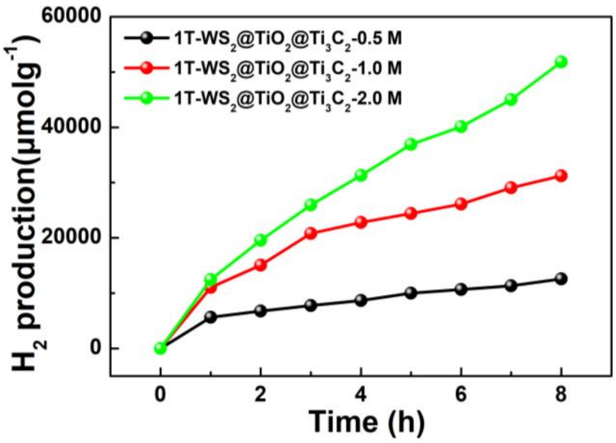

Fig. S11 The photocatalytic H_2 production of the samples with different molar amount of HCl (0.5, 1.0, and 2.0 M) under simulated sunlight illumination

In order to study the influence of three compositions in 1T-WS_2@TiO_2@Ti_3C_2 on the photocatalytic properties of composites, it is necessary to explore whether the performance can be improved by further increase the proportion of addition. So in 1T-WS_2@TiO_2@Ti_3C_2 composites, the content of WS_2 (15 wt% WS_2) is kept unchanged. We further changed the ratio between Ti_3C_2 and TiO_2 by changing the molar amount of HCl (0.5, 1.0, and 2.0 M), so as to explore the photocatalytic performance of 1T-

WS_2@TiO_2@Ti_3C_2 composites. As shown in **Fig.** S11, with the increase of molar amount of HCl from 0.5 to 2.0 M, an increase in the photocatalytic performance of 1T-WS_2@TiO_2@Ti_3C_2 composites is discovered. It can be concluded that the three compositions in 1T-WS_2@TiO_2@Ti_3C_2 work together to improve the photocatalytic performance of the composite.

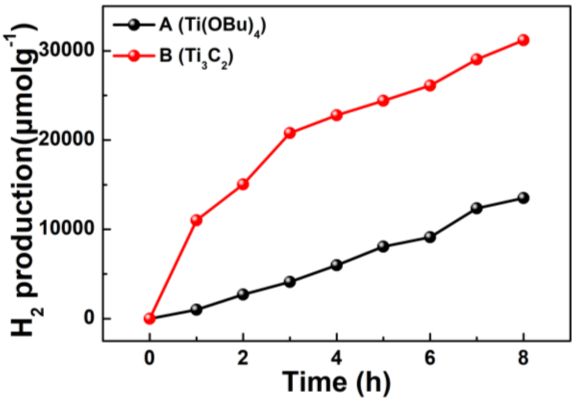

Fig. S12 The photocatalytic H_2 production of the samples with different titanium sources under simulated sunlight illumination

Considering the in-situ loading of TiO_2 nanosheets has poor controllability and has a certain influence on the structure and properties of MXene nanosheets, therefore we synthesis of TiO_2 nanosheets by foreign titanium sources. TiO_2 nanosheets (foreign titanium sources) is synthesized as follows: 1.0 mL $Ti(OBu)_4$ was slowly dropped into HCl (18 mL, 5 M) solution. After the solution was stirred for 30 min, 0.35 mL HF was added to the mixed solution. After another 5 min stirring, the solutions were transferred into Teflonlined stainless-steel autoclaves with a total volume of 25 mL. The hydrothermal synthesis was conducted at 180 °C for 4 h in an electric oven. To synthesis of 1T-WS_2@TiO_2@Ti_3C_2 composites (foreign titanium sources), 24 mg WCl_6, 9 mg TAA, 80 mg Ti_3C_2 and 20 mg TiO_2 nanosheets (foreign titanium sources) were dissolved in 50 mL DMF to form a transparent solution. After stirred for 60 min, the solution was transferred into a 100 mL Teflon-lined stainless-steel autoclave, which was hydrothermally treated at 200 °C for 24 h. After naturally cooling down to room temperature, the reaction solution was collected by vacuum filtration, and the resulting 1T-WS_2@TiO_2@Ti_3C_2 composites (foreign titanium sources) were washed with distilled water several times, and dried in vacuum oven at 60 °C for 12 h. The photocatalytic performance of 1T-WS_2@TiO_2@Ti_3C_2 composites (foreign titanium sources) was evaluated using H_2 evolution under simulated sunlight irradiation in an aqueous acetone solution at room temperature (**Fig.** S12). Compared with in-situ loading of TiO_2 nanosheets, the foreign titanium sources do not improve the photocatalytic performance of 1T-WS_2@TiO_2@Ti_3C_2 composites, which may be caused by the non-close contact between TiO_2 and Ti_3C_2 caused by the foreign titanium sources.
